# Failure to thrive in toddlers with lack of interest in eating and food and their cognitive development during later childhood

**DOI:** 10.3389/fped.2023.1179797

**Published:** 2023-08-29

**Authors:** Irene Chatoor, Rebecca Begtrup, Iris Yao Cheng, Laura Vismara, Lauren E. Webb, Loredana Lucarelli

**Affiliations:** ^1^ Department of Psychiatry and Behavioral Sciences, George Washington University, Washington, DC, United States; ^2^ Children's National Hospital, Washington, DC, United States; ^3^ Centers for Disease Control and Prevention (CDC), Atlanta, GA, United States; ^4^ Department of Pedagogy, Psychology, Philosophy, University of Cagliari, Cagliari, Italy; ^5^ Department of Psychology, Hofstra University, Hempstead, NY, United States

**Keywords:** failure to thrive, ARFID, cognitive development, longitudinal study, parenting

## Abstract

**Background:**

Experiencing Failure to Thrive or malnutrition in early years has been associated with children later displaying low Intelligence Quotient (IQ). The current study's aim was to examine whether Failure to Thrive in Toddlers with Lack of Interest in Eating and Food, a subtype of Avoidant/Restrictive Food Intake Disorder as defined by DSM-5, which has also previously been identified as Infantile Anorexia (IA), was associated with poor cognitive development outcomes during later childhood.

**Methods:**

The IQs and growth parameter of 30 children (53% female) previously diagnosed and treated for IA at 12 to 42 months of age, were reevaluated at a mean age of 10.0 years (SD = 2.1 years) and compared to 30 matched control children. Children's growth was assessed using Z-scores and their cognitive development was measured using the Wechsler Intelligence Scale for Children-4th Edition.

**Results:**

None of the growth parameters were significantly related to IQ. Further, IQ scores of children previously diagnosed with IA and control children were not significantly different. However, the education level of children's fathers had a significantly positive effect on IQ.

**Conclusions:**

Our study highlights the disjunction between growth parameters and IQ within our sample. Overall, our findings suggest that the primary target of intervention for these children should be the parent-child conflict around the feeding relationship, rather than a focus on the child's weight itself. Finally, our results confirm the relevance to include fathers in the intervention of these children.

## Introduction

Failure to thrive (FTT) is a condition that describes children with growth deficiency as evidenced by failure to make expected weight/height gains or by significant weight loss. Previous studies found that childhood FTT/malnutrition are associated with low Intelligence Quotient (IQ) (1–3). Further research demonstrated confounders to this link, such as mother's IQ, maternal education, maternal-child interactions, and socio-economic status (4). Infantile Anorexia (IA) is the most common type of feeding disorder associated with malnutrition. Diagnosis of IA is based on the DC:0–3R criteria (5) and was redefined by the DSM-5 (6) as a criterion (i.e., “apparent lack of interest in eating or food”) of Avoidant/Restrictive Food Intake Disorder (ARFID). For the purpose of the current study, IA will be the term utilized. Children with IA experience low hunger drive and rapid satiety. They often prefer to play and socialize instead of eat and may meet criteria for FTT. This is a non-organic form of FTT (i.e., lacks an anatomic cause), but the malnutrition is not associated with maternal deprivation or neglect (7–10). Therefore, growth deficiency can be examined as it relates to cognitive outcomes separate from the confounders of severe psychosocial risk factors. Previous research emphasized the importance of weight restoration in those with FTT to prevent adverse long-term effects on IQ. This would appear relevant for children with IA, as it can result in FTT and would suggest a negative effect on IQ. However, increased pressure to restore weight often leads to increased parental anxiety (9, 10). Anxious parenting heightens distress in the child leading to further food refusal, which causes more parental anxiety, creating a vicious, enduring, and self-perpetuating cycle which further exacerbates the child's malnutrition (11). These patterns may be observed during mealtime when strong conflict may emerge as manifested by the child's food refusal and the parent's negative affect and/or comments about the child's refusal to eat (5, 12). It is unclear whether those with IA and FTT have lower IQs. The current paper hypothesizes that IQs of children diagnosed with IA during toddlerhood are not significantly correlated to their weights at either the times of diagnosis or follow-up. With the pressure for rapid weight gain removed, the focus can instead shift to resolving parent-child conflict related to eating, as described in a previous paper by Chatoor, Ganiban, Hirsch, Borman-Spurrell, and Mrazek (12).

## Specific aims

The specific aim of the current study is to examine whether malnutrition in children within a low psychosocial risk sample, diagnosed and treated for IA during the first three years of life has any association with their cognitive development during later childhood.

## Methods

### Participants

Participants from the current follow-up study include 60 children ranging in age from 7 to 13 years. Thirty of the children previously participated in an initial treatment study completed six years prior, and were members of the initial treatment Infantile Anorexia study group. The initial study included 70 toddlers ranging in age from 12 to 42 months, all of whom were recruited from a Feeding Disorders Clinic at a University Hospital in the United States, where they were seeking treatment for Infantile Anorexia (IA). The diagnosis of IA was made by two independent clinicians (*k* = 0.93) at the time of the initial study. Following the publication of the DSM-5 (6), the children originally diagnosed with IA and enrolled in the initial study met criteria for ARFID via the criterion “apparent lack of interest in eating or food”. The initial treatment study spanned 5 years. Parents of these children who completed the treatment study were invited to participate in the current study. Of note, in the initial study, the toddlers with IA were assigned randomly to one of two interventions.

The interventions were inspired by a transactional model that comprises three main factors for IA: infant's difficult temperament, maternal psychopathology, and intrusive, controlling or permissive parenting styles. Children with IA have higher levels of physiological arousal and more difficulty down-regulating their arousal; consequently, they demonstrate interest in everything but eating. The limited food intake leads to poor growth which triggers parents of these infants and young children to become quite anxious. At times these parents will become so desperate that they resort to force-feeding the children. This tension increases for all parties and often results in making mealtimes very stressful for the entire family.

The second intervention was aimed at explaining the infant's temperament (i.e., high arousal and difficulty turning off excitement to allow for eating and/or sleeping, difficulty in recognizing hunger and fullness cues, and difficulty with self-soothing when upset), addressing the parents’ backgrounds (i.e., eating history and difficulty with limit setting), and providing specific feeding guidelines and time-out procedure. The goal was to remove conflict in the parent-child relationship, facilitate the child's recognition of hunger and fullness cues, work on limit setting, and teach the child self-calming techniques (13–15).

Brief details regarding the two treatment conditions from the initial study are provided below, and further information can be found in Chatoor (13–15).

Initial Study Intervention I—Full Treatment: During the first 2 sessions, the child's temperament, the parents' limit setting, and eating behavior were discussed. The following 4 sessions centered on providing psychoeducation to parents regarding feeding guidelines and a time-out procedure to teach the children self-calming.

Initial Study Intervention II—Partial Treatment [Control Condition]: During the first two sessions, the parents were asked to complete questionnaires and were shown an educational videotape. The following 4 sessions were the same as Intervention I.

Both groups did comparatively well at 1 year's follow up, showing a significant decrease of both mother-toddler conflict and struggle for control during feeding, and improving the child's growth (16).

**Figure 1:**
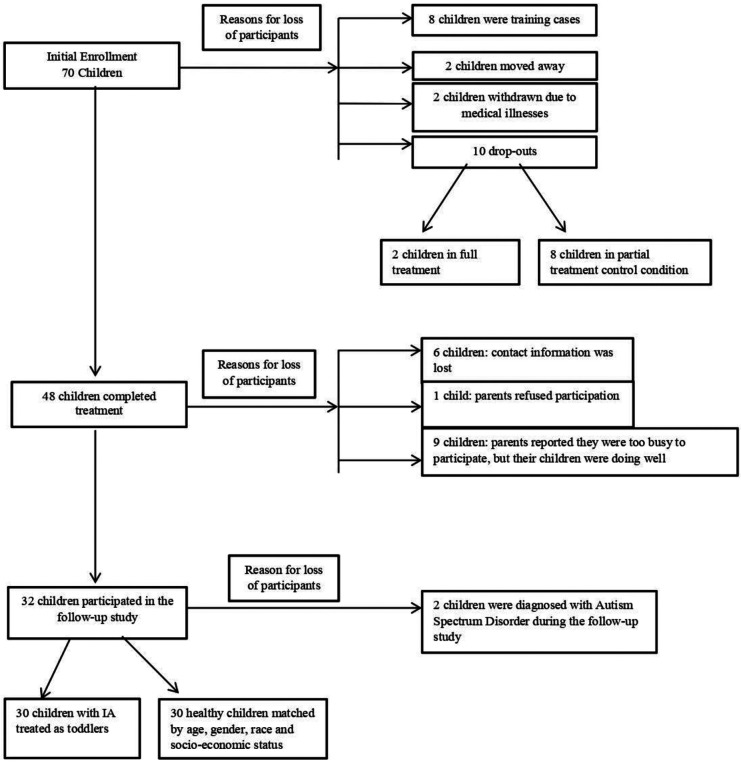
Sample enrollment and drop outs.

Figure 1 describes how the initial sample of 70 children who participated in the treatment study shrank to 30 children who were seen for the follow-up study. Children who dropped out at follow-up and those who were seen for follow-up did not differ in any baseline sociodemographic measure.

In the initial study, four trained therapists administered the treatment. The first 8 children were considered training cases and were excluded from the data analysis. In addition, 2 children moved away after treatment, another 2 children were withdrawn from treatment due to medical illnesses, and 10 additional children dropped out of the research study seeking individualized treatment (eight of whom were from the partial treatment control condition and two of whom were in the full treatment condition). None of these children were included in the data analysis. The remaining 48 children completed the treatment (Figure 1).

Before the follow-up study, the research assistant attempted to contact the parents of the 48 children who completed the initial treatment. Of the 48 children, only 32 children were able to participate in the follow-up study. Due to unreported changes in families' contact information, 6 families could not be reached. Another 9 children did not participate in the follow-up study because their parents stated that they were too busy, but that their child was doing well. One parent was unhappy about the treatment, and refused to participate in the follow-up study. Of the 32 children who participated in the follow-up study, 2 children were diagnosed with Autism Spectrum Disorder and were not included in the data analysis.

Because of the small sample size, the 2 treatment groups were collapsed for the follow-up study.

These 30 children were matched by age, sex, race and educational level of the parents with 30 healthy children without any history of eating problems (control group; CG) to serve as a methodological control for the IQ assessment. These children were recruited via posters and questionnaires from a pediatric ambulatory clinic.

### Measures

#### Anthropometric measures of growth deficiency

The children's growth status was assessed using their weight and height measurements. These measurements were taken by the nurse practitioner and recorded on standard NCHS growth charts (17).

#### Z-scores

The z-score was used to define their weight and height. A z-score is a method used to calculate the probability of a score occurring within the normal distribution. A z-score of zero is considered the average. Mild, moderate, or severe risk of malnutrition respectively corresponds with −1.0 to −1.99, −2.0 to −2.99, and less than −3.0 for z-score ranges. High average, superior and high superior z-score respectively corresponds to +1.0 to +1.99, +2.0 to +2.99, and +3.0.

#### The Wechsler Intelligence Scale for Children—4th Edition

The Wechsler Intelligence Scale for Children-4th Edition (WISC-IV) (18) was used to measure the child's intellectual and cognitive abilities. The WISC-IV provides four index scores: verbal comprehension, perceptual reasoning, working memory, and processing speed. This is a widely used measure that has high internal consistency and construct validity. The standard score ranges for the Wechsler Intelligence Scale are as follows: 70 to 79 is below average, 80 to 89 is low average, 90 to 109 is average, 110 to 119 is high average, 120 to 129 is superior, and 130 and greater is very superior. The WISV-IV was utilized because the WISC-V was not yet published when data was collected.

### Procedures

Participants' weight and height values measured at initial assessment during the treatment study were retrieved for the follow-up study. During the follow-up study, all children were weighed and measured again by a nurse practitioner, and the WISC-IV was administered by a psychologist who was blinded to their group assignment. The weights, heights, and The Full-Scale Intelligence Quotient (FSIQ) scores of children in the IA group were compared to the weight, height, and FSIQ scores of children in the Control Group (CG) who were matched by age, gender, and socio-economic status.

The study protocol was reviewed and approved by the IRB, and all parents signed informed consent forms.

### Statistical analysis

Descriptive statistics for the social demographics, height, weight, and FSIQ measure were calculated and reported as means and frequencies. A paired t-test was conducted to examine if the changes from the baseline to follow-up for the growth parameters were significant based on Type I error of 0.05. A Pearson correlation coefficient was calculated for the relationship between the growth parameters and the IQ measure. A t-test was also used for comparing the IA and control groups for the IQ measure. Finally, a linear regression model was conducted to compare the IQ of the IA group and CG, controlling for mother's education and father's education. Statistics software R was used for the analyses.

## Results

Demographic information for the 30 children in the IA group and the 30 healthy children in the CG are described in Table 1. Children in the IA group had a mean age 10.0 years (SD = 2.1 years, range = 6.4–13.1 years). Additionally, 53% of the IA group was female and the mean number of years of mother's and father's education was 17 years (SD = 0.5). The children in the control group were matched with the IA children and had similar distributions for social demographics. Of note, the mothers of the children in the CG group had one year more education, while the fathers had 1.3 years more education than the parents in the IA group.

**Table 1:** Sample characteristics.

Variable	IA children (*N* = 30)	CG children (*N* = 30)
Age: Mean (SD)	10.0 (2.1)	10.3 (1.6)
Age: Range	6.4–13.1	6.5–13.6
Gender		
Female, *N* (%)	16 (53)	18 (60)
Male, *N* (%)	14 (47)	12 (40)
Years of mother's Education: Mean (SD)	17.1 (0.5)	18.1 (0.5)
Years of father's Education: Mean S (SD)	17.4 (0.5)	18.7 (0.4)

**Figure 2:**
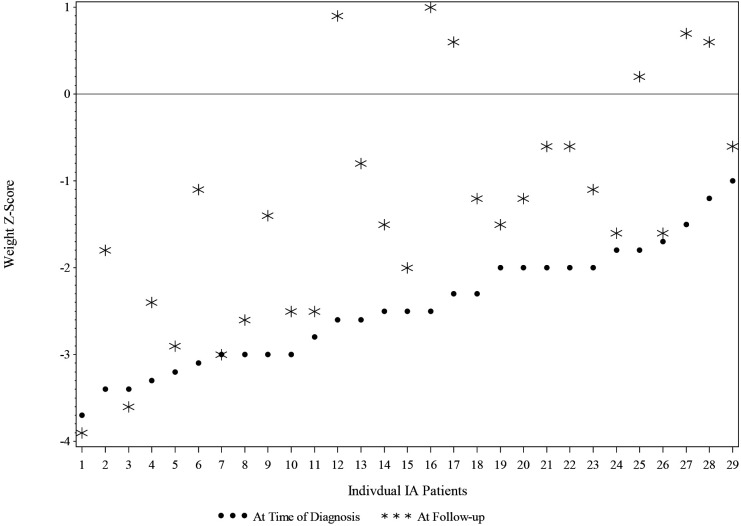
Weight Z-score at time of diagnosis and at follow-Up for IA children (*N* = 30).

Changes in weight and height in the IA group's children between the first study pretreatment (Time point 1; T1) to the post-treatment current follow-up (Time point 2; T2) are reported in Table 2 and Figure 2. Mean weight z-scores increased significantly from T1 to T2. At T1, children in the IA group had a mean z-score of −2.46 (SD = 0.69), whereas at T2, they had a mean z-score of—1.31 (SD = 1.33). The mean of the increase of the z-score was 1.14 (95% CI: 0.76–1.53, *p* = <0.0001).

**Table 2:** Descriptive statistics for IA's children growth parameters at T1 and at T2, and comparison between these Two time points.

Growth variable	Baseline (*N* = 30)	Follow-up (*N* = 30)	Change T2–T1	*p*-value*
	Mean (SD)	Mean (SD)	Mean (95% CI)	
Weight z-score	−2.46 (0.69)	−1.31 (1.33)	1.14 (0.76, 1.53)	<0.0,001
Height z-score	−0.97 (0.81)	−0.99 (1.12)	−0.003 (−0.309, 0.302)	0.9,817

* Paired *t*-test was used.

**Figure 3:**
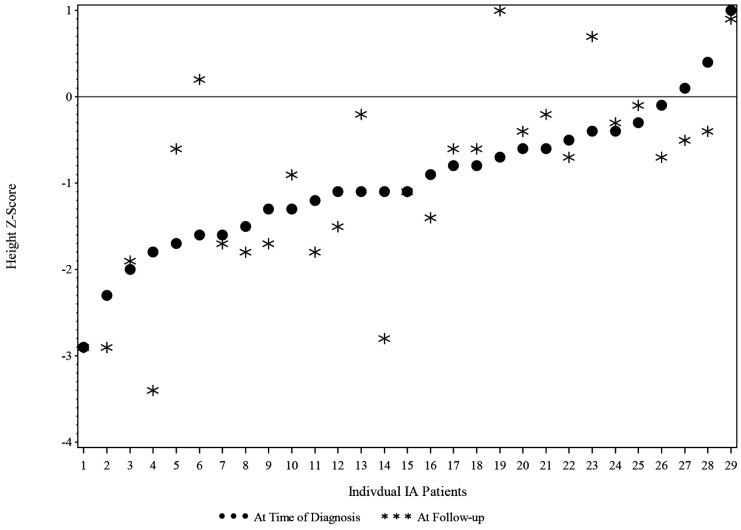
Height Z-score at time of diagnosis and at follow-Up for IA children (*N* = 30).

The IA group height z-score at T1 was grouped into 2 categories: equal to or less than −1.0 and greater than −1.0. The changes in the height z-score from T1 to T2 were not significant for children in the IA group, as shown in Figure 3.

The Pearson correlation coefficients between the children of the IA group's z-scores and FSIQ at T1 and at T2 are reported in Table 3. None of the measures were significantly associated with FSIQ.

**Table 3:** The correlations between IA's z-scores and FSIQ at T1 and at T2 (*N* = 30).

Variable	Pearson Correlation Coefficient (r)	*p*-value
Weight z-score at baseline	−0.16	0.43
Height z-score at baseline	0.25	0.21
Weight for height z-score at baseline	−0.33	0.10
Weight z-score at follow-up	−0.07	0.71
Height z-score at follow-up	0.17	0.37
BMI z-score at follow-up	−0.21	0.28

**Figure 4:**
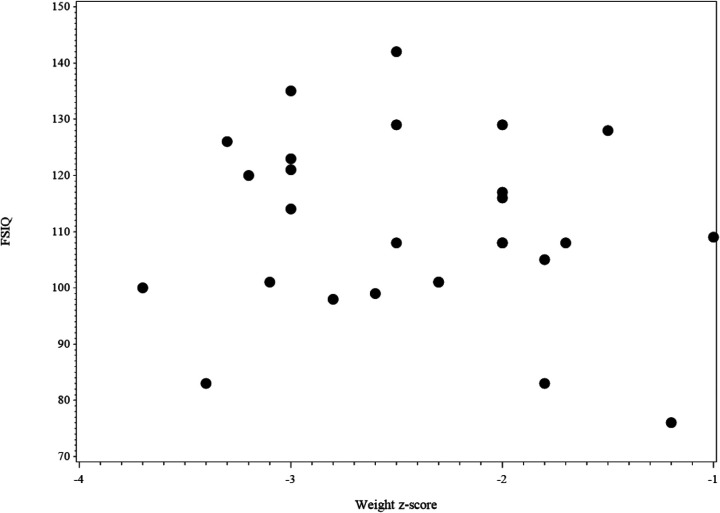
The scatter plot of FSIQ score at follow-up vs. weight z-score at the baseline for IA children (*N* = 27). 2 missing values in FSIQ and 1 missing value in weight z-score, 3 missing values in the figure.

**Figure 5:**
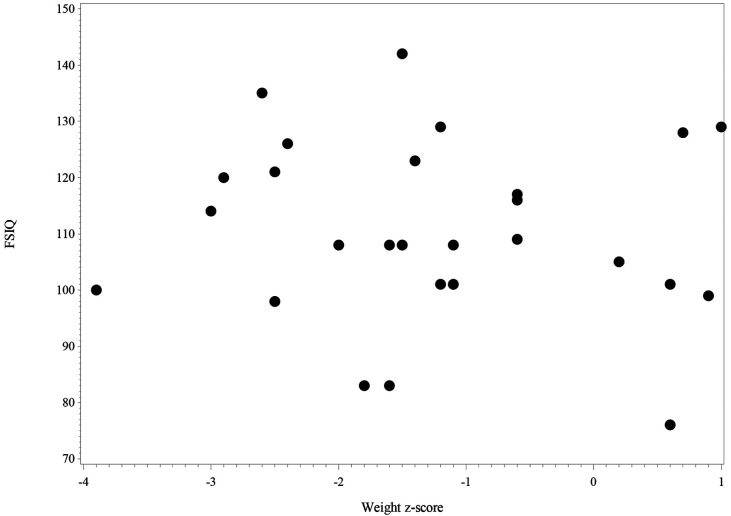
The scatter plot of FSIQ score at follow-up vs. weight z-score at follow-up for IA children (*N* = 28). 2 missing values in FSIQ and no missing value in weight z-score, 2 missing values in the figure.

Figure 4 shows the scatter plot of FSIQ at follow-up vs. the Weight z-score at T1. The scatter plot (*N* = 27) shows the range of FSIQ was 76–142 and the range of the weight z-score was −3.7–−1.0. The dots in the plot didn't show any linear pattern that matches with the linear relationship; thus, the correlation was not significant. Also, no significant correlation was found in relation to the scatter plot of FSIQ at follow-up vs. the Weight z-score at T2 (Figure 5).

A comparison of FSIQ scores from children in the IA group and children in the CG is reported in Table 4. Table 5 displays results from the linear regression model analysis that was used to test the differences in FSIQ between the two groups, controlling for mothers' and fathers' education levels. The analysis confirmed that although children from the IA group had a somewhat lower FSIQ than children in the control group, there was no statistically significant difference (*β* = −3.84, *p*-value = 0.28). However, the fathers in the control group had 1.3 years more education, which had a significantly positive effect on overall FSIQ (*β* = 1.88, 95% CI = 0.33–3.42, *p*-value = 0.019). An increase of father's education by one year, resulted in a significant increase of 1.88 in the average FSIQ.

**Table 4:** Descriptive statistics of the two groups of children at T2.

	IA's Children (*N* = 30)	CG Children (*N* = 30)	*p*-value*
FSIQ: mean (SD)	110.2 (15.9)	116.9 (10.5)	0.0,662

* *T*-test.

**Table 5:** Linear regression result for FSIQ at the follow-up.

Predictor	Effect Estimate (*β*)	95% CI for Effect	*P*-value
Group			
History of IA	−3.84	−1.09,0.28	0.282
Control Group	–	–	–
Years of mother's education	−0.37	−0.53, 0.60	0.601
Years of father's education	1.88	0.33, 3.42	0.019

-: The reference group for a categorical predictor.

## Discussion

The main aim of the study was to examine whether malnutrition in children diagnosed with and treated for ARFID IA during the first three years of life had any association with their cognitive development during later childhood in a sample of low psychosocial risk families.

Previous research has shown that malnutrition and FTT negatively influence children's cognitive development (1, 2), however, most of these studies involved children recruited from developing countries. The environmental context of a developing country is characterized by multiple concurrent risk factors that may interfere with the child's cognitive performance. Indeed, nutritional status has been identified as a factor impacting children's neuropsychological development (19). Still, the quality of a child's cognitive performance is also determined by numerous psychosocial factors. Among these, mother's education is a significant variable in the cognitive development of children (18, 20–22) Maternal IQ, which is associated with marital status, income level, and quality of the home environment, is also among the strongest predictors of children's cognitive performance (21). Moreover, relevant literature has highlighted that increased psychosocial risk factors in families are associated with lower child cognitive performance (22–24).

The current follow-up study revealed that none of the child's growth parameters were significantly related to their FSIQ. These findings are in line with Chatoor et al's (4) previous study, which purported that mother-toddler interactions, maternal education level, and SES level explained more unique variance in Mental Developmental Index scores than nutritional status.

No significant difference was found between the FSIQ of children in the IA group and the children in the CG. Of note, however, father's education showed a significantly positive effect on overall FSIQ. Previous studies have shown that fathers' education and income are associated with child cognitive achievements, and fathers' education is associated with the quality of mother-child relationship (25, 26). Moreover, fathers who are more educated and economically secure seem to have more positive interactions with their children (26). Therefore, results of the current study underscore the importance of considering fathers in studies on child development, as they offer important protective factors alongside the unique, independent contribution from mothers (27–29). Undeniably, several advantages of paternal engagement in intervention have been demonstrated, such as family economic contributions, better mother-infant attachment quality, academic success, and lower child's externalizing and internalizing problems (30, 31).

## Limitations

The current follow-up study possesses some limitations, given the multifactorial nature of malnutrition and cognitive development. First, the study has not included the assessment of other variables, such as parental psychopathology, child's school adjustment, and family adversities, all of which may represent mediating/moderating factors in the developmental trajectories of children with IA. Additional information on environmental factors should be included in future studies to fully understand their role in children's later cognitive performance. Finally, results should be replicated using a larger sample for generalizability of the findings within low-psychosocial risk samples.

## Conclusion

Although the current study does not allow the establishment of cause and effect between nutrition and cognitive development, overall, its findings suggest that the primary treatment target for children with ARFID IA in low psychosocial risk families should be parent-child conflict around the feeding relationship, as opposed to trying to rapidly increase the children's weight. Current findings demonstrated that children's cognitive levels are not directly impacted by their weight. Of note, other studies have shown that poor cognitive performance in children is associated with possessing parents who are less sensitive and responsive to the emotional needs of their children or who interact with less positive emotionality (4, 27, 32, 33).

## Implications for practice

Feeding is a complex transactional process between the child and the parent. Several studies have shown that mothers of children with ARFID IA are more intrusive (9, 34, 35) and demonstrate greater conflict and struggle for control during feeding interactions with their children (9, 35, 36) than mothers of children without ARFID IA. Confronted with children's food refusal, some mothers may become very anxious and force-feed their children. The more they force their children to eat, the more the child rejects the food, generating a negatively reinforcing cycle of control between the child's food refusal and the parents' anxiety (9, 34, 36). It is critical that intervention for families focuses on parent-child conflict around the feeding relationship by helping parents change their behavior, ultimately changing the child's behavior.

## Data Availability

The data are not publicly available due to information that could compromise the privacy of research participants. Requests to access the datasets should be directed to YC, ycheng5@yahoo.com.
